# Intraligamentary Anesthesia in Pediatric Patients: Is It an Effective Technique? A Systematic Review and Meta-Analysis

**DOI:** 10.3390/jcm15051828

**Published:** 2026-02-27

**Authors:** Claudia Salerno, Silvia Cirio, Aesha Allam, Marta Mazur, Maria Grazia Cagetti

**Affiliations:** 1 Department of Biomedical, Surgical and Dental Sciences, University of Milan, 20122 Milan, Italy; claudia.salerno@ymail.com (C.S.); silvia.cirio@unimi.it (S.C.); aesha.allam@unimi.it (A.A.); 2 Interdisciplinary Department of Wellbeing, Health and Environmental Sustainability—BeSSA Department, Sapienza University of Rome, 02100 Rieti, Italy; marta.mazur@uniroma1.it

**Keywords:** intraligamentary anesthesia, pediatric dentistry, local anesthesia, computer-controlled local anesthetic delivery, randomized controlled trials

## Abstract

**Background**: Effective pain control is fundamental in pediatric dentistry. Supraperiosteal infiltration (SPA) and inferior alveolar nerve block (IANB) are the most used local anesthesia (LA) techniques. This review evaluated the available evidence on intraligamentary anesthesia (ILA) to assess its efficacy, safety, and viability as an alternative to conventional techniques. **Methods**: The review protocol was registered in PROSPERO (CRD420261284494) and conducted in accordance with PRISMA guidelines. Three databases were searched for RCTs published in English after 2000 involving children. Studies that compared ILA, delivered via either traditional or computer-controlled systems (CC-ILA), with other LA techniques were included. Risk of bias was assessed using the Cochrane’s RoB 2.0 tool. Meta-analysis was performed using a random-effects model with Stata/SE 18.0. **Results**: The database search yielded 347 records; after duplicate removal, 153 articles were screened. Thirty-four papers were assessed, of which thirteen studies were included, and three were retained for the meta-analysis. Significantly lower pain perception and improved physiological parameters were reported with ILA compared with IANB. CC-ILA demonstrated greater efficacy and reduced procedural discomfort than conventional ILA. Patients favored CC-ILA over IANB (68.0% vs. 32.0%). Postoperative lip biting occurred more frequently following IANB and CC-SPA than after ILA. Overall risk of bias was low. Meta-analysis revealed no significant difference in pain perception between ILA and IANB (z = −0.26; *p* = 0.79). **Conclusions**: ILA, particularly CC-ILA, appears to be an effective, safe, and well-tolerated technique and may be considered a valid anesthetic option in pediatric dentistry. The review did not receive any funding.

## 1. Introduction

Pain management during dental procedures represents a central component of clinical practice, particularly in pediatric dentistry. The need for local anesthesia (LA) in pediatric dental practice reflects the persistent burden of dental caries in childhood [1]. The administration of LA constitutes a critical phase of the clinical appointment. Although it may be perceived as stressful by young patients, LA is essential for ensuring adequate pain control and improving patient comfort. An ineffective LA can compromise patient cooperation and lead to dental fear and anxiety, increasing the need for more invasive behavior management strategies to perform successful dental treatment [2,3,4]. Although fear of sharp objects, such as needles, may be considered an innate response, pain associated with LA primarily results from mechanical trauma caused by needle insertion and tissue distension following anesthetic injection. The adoption of an appropriate anesthetic technique, tailored to the patient’s age, is therefore crucial in modulating pain perception [5].

The most used LA techniques in pediatric dentistry include supraperiosteal infiltration and the inferior alveolar nerve block (IANB), performed using conventional dental syringes, disposable cartridges, and standard needles. IANB enables anesthesia of multiple teeth within the same quadrant, making it particularly useful in various clinical situations [6]. However, reported IANB failure rates range from 31% to 81%, mainly due to individual anatomical variations and inaccuracies in needle placement [7,8]. The sensation of soft tissue anesthesia, common to both techniques, may be perceived negatively by pediatric patients who often have difficulty distinguishing between pain and the anesthetic effect. This may reduce cooperation in the post-injection phase. Moreover, the duration of soft tissue anesthesia, which often exceeds pulpal anesthesia, may increase the risk of postoperative soft tissue injuries, such as accidental lip or cheek biting, particularly in pediatric or special needs patients [9].

Intraligamentary anesthesia (ILA), or injection into the periodontal ligament, has been proposed as an alternative technique for single-tooth anesthesia (STA). Although similar approaches were described in the early 20th century, clinical adoption was initially limited due to the lack of suitable dental instruments. It was only with the introduction of specialized high-pressure syringes in the late 1970s and early 1980s that ILA gained wider acceptance in modern dental practice [5]. This technique allows rapid onset of anesthesia with a short needle and a reduced volume of anesthetic in the periodontal ligament, and typically provides tooth anesthesia lasting about 1 h, depending on the agent used [10]. A primary advantage of ILA is the absence of anesthesia of oral soft tissues, such as the lips and tongue, reducing the risk of postoperative injuries. Furthermore, ILA can serve as a supplemental technique when conventional methods are insufficient, such as in cases of incomplete anesthesia during endodontic or extraction procedures. Nonetheless, certain limitations have been reported, including transient bacteremia, postoperative discomfort, and the subjective sensation of “tooth elevation” [11]. Histological studies in animal models indicate that any ligament alterations induced by injection are generally minor, localized, and reversible, without permanent damage to the surrounding tissues [12]. Comparative analyses suggest that while overall anesthetic efficacy may be similar, ILA may be associated with higher injection-related pain than conventional infiltration techniques due to the higher injection pressure applied [13].

In this context, assessing pain perception is central, as it represents a key clinical outcome in selecting anesthetic techniques for children. Self-report pain scales remain the gold standard in pediatric populations, but inherently provide subjective data, which should be complemented by objective physiological or behavioral measures for a more reliable evaluation. Commonly used instruments include the Visual Analog Scale (VAS), Wong–Baker FACES Pain Rating Scale (WBFPRS), Faces Pain Scale–Revised (FPS-R), Numeric Rating Scale (NRS), and McGill Pain Questionnaire [14,15]. Non-invasive devices, such as pulse oximeters, allow monitoring of heart rate and arterial oxygen saturation, offering reliable, cost-effective, and easily applicable tools for indirect physiological pain assessment.

Since the late 1990s, innovative delivery systems for LA have been introduced, evolving to improve ease of use, predictability, and patient comfort. Among these, computer-controlled local anesthetic delivery systems (CCLAD) represent a significant technological advancement and can be used for LA delivery in different techniques including computer-controlled intraligamentary anesthesia (CC-ILA) and computer-controlled inferior alveola nerve block (CC-IANB) [16]. CCLAD devices employ microprocessors to precisely control flow, modulating pressure and injection speed based on tissue resistance. This allows gradual anesthetic diffusion, reducing tissue distension and injection-related pain [17]. Clinical studies show that CCLAD may improve patient behavior and cooperation, positively impacting the overall clinical experience [18]. However, the benefits may vary with age, with adults typically experiencing greater pain reduction and more predictable efficacy compared with children [19]. In light of this evidence, ILA, particularly when administered via computer-controlled systems, represents a potential alternative to conventional LA methods in pediatric dentistry. Accordingly, this systematic review was conducted to select and critically analyze published studies on ILA in pediatric dentistry to assess clinical efficacy, safety, and its potential role as an alternative to conventional techniques.

## 2. Materials and Methods

### 2.1. Protocol Registration

The systematic review protocol was registered at the International Prospective Register of Systematic Reviews (PROSPERO), registration number: CRD420261284494. The review adhered to the methodology outlined in the Cochrane Handbook of Systematic Reviews. It conformed to the guidelines set forth by the Preferred Reporting Items for Systematic Reviews and Meta-Analysis (PRISMA) [20]. PRISMA checklist is provided in Supplementary Table S1.

### 2.2. PICOs Questions

The main inquiries addressed in the present review were devised and stated: “What is the efficacy and safety of ILA in children and adolescents undergoing dental treatment compared with traditional local anesthetic techniques? Furthermore, are there any differences between traditional ILA and ILA performed using computerised techniques?” Elements of the PICOs model were the following:

P (Participants): Children of either sex younger than 18 years;

I (Intervention): ILA delivered either using a conventional technique or a computer-controlled delivery system for any type of dental treatment;

C (Comparison): Conventional local anesthesia (e.g., IANB), administered using either traditional or computer-controlled delivery systems; in addition, studies employing any alternative techniques for delivering ILA were also considered;

O (Outcomes): The primary outcome is pain/discomfort perception; the secondary outcomes are anxiety, need for additional anesthesia, and postoperative complications;

S (Studies): Randomized controlled trials (RCTs).

### 2.3. Information Sources and Search Strategy

Three databases (PubMed, Embase, and Scopus) were searched from January 2000 to 17 December 2025. The search strategy was initially developed for PubMed using keywords and MeSH terms related to ILA administration in children and then adapted for the other databases. Search strings used for each database are displayed in the Supplementary Table S2. Cross-referencing was also performed using the reference lists of full-text papers.

### 2.4. Study Selection, Eligibility Criteria, and Data Extraction

Before the review process, eligibility criteria were predefined. A preliminary screening exercise was conducted prior to formal screening to ensure correct and consistent application of the eligibility criteria. Two reviewers (S.C., M.M.) underwent training, and a third reviewer (M.G.C.) was designated to resolve disagreements. After duplicate records removal, the formal screening began, and the two authors assessed the records based on title and abstract; disagreements were resolved by discussion, and when this was not possible, the third author was consulted. Subsequently, the same two authors proceeded to full-text analysis; disagreements were resolved by debate or involvement, where needed, with the same third author. Inter-reviewers’ agreement was assessed at both stages using Cohen’s Kappa value.

Studies were evaluated based on the following inclusion criteria: RCTs conducted on subjects younger than 18 years, availability of the full text in English, publication year after 2000, studies that compared ILA performed with either traditional or computer-controlled delivery systems with any other anesthetic techniques, and studies that compared ILA administered through any other technique.

Data extraction was performed independently by the two reviewers (C.S., A.A.). The following data were collected and inserted in an Microsoft Excel® (Microsoft Corporation, Redmond, WA, USA) extraction form: bibliographic information (authors, publication year, journal, country), study design, study population (age range, number of participants assigned to each group, male–female ratio), type of intervention, and assessed outcome, along with the results of each group.

### 2.5. Risk of Bias Assessment

The risk of bias assessment was carried out independently by two reviewers (A.A., S.C.) using the Cochrane Collaboration’s RoB 2.0 [21]; disagreements were resolved by a third author (C.S.). The tool comprises five domains in which risk of bias is evaluated: bias arising from the randomization process, bias due to deviations from intended interventions, bias due to missing outcome data, bias in the measurement of the outcome, and bias in the selection of the reported result. For each domain, the risk of bias was judged as “low risk”, “some concerns”, or “high risk”. Assessment of each domain was based on a series of signaling questions, which could be answered as “Yes,” “Probably yes,” “No information,” “Probably no,” or “No”. The overall risk of bias for each study was considered “low” if all domains were judged as low risk, “some concerns” if at least one domain raised some concerns, and “high” if at least one domain was judged as high risk or if multiple domains raised some concerns. The Rob-vis tool was used to generate a visualization of the risk of bias across the included studies [22].

### 2.6. Meta-Analysis

Based on the studies included in the systematic review, the feasibility of performing a quantitative meta-analysis was carefully assessed. Most of the available studies showed substantial clinical and methodological heterogeneity, particularly regarding the type of local anesthesia compared and the outcomes assessed. Due to this heterogeneity, a global meta-analysis including all studies was considered inappropriate. Therefore, the quantitative synthesis was restricted only to studies that were homogeneous in terms of outcome and intervention. This was possible only for studies evaluating pain perception using the same outcome measure (VAS) and directly comparing ILA with IANB. For each study, mean values, standard deviations, and sample sizes were extracted. When standard deviations were not explicitly reported, they were calculated from the corresponding 95% confidence intervals. Stata/SE 18.0 for Mac (Intel 64-bit) (StataCorp LLC, College Station, TX, USA) was used for meta-analysis. A random-effects meta-analysis using the restricted maximum likelihood (REML) method was performed to account for between-study variability. Effect sizes were expressed as Cohen’s d with 95% confidence intervals. Statistical heterogeneity was assessed using the Q test and the I^2^ statistic. Publication bias was visually explored using a funnel plot.

## 3. Results

### 3.1. Databases Search Results

**Figure 1:**
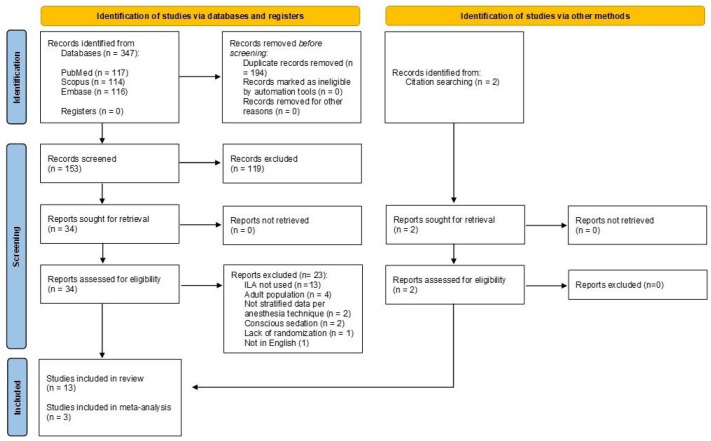
PRISMA flowchart of the screening process.

The database search results are presented in the flowchart shown in Figure 1. The search yielded a total of 347 records. After removal of duplicates, 153 records were screened based on title and abstract, and 119 were excluded. Consequently, 34 records were deemed eligible and progressed to full-text evaluation. Thirteen records were excluded as they did not report the use of ILA; four records reported intervention on the adult population, two records lacked stratified data per local anesthesia technique, two records reported the use of ILA during conscious sedation, one record lacked randomization, and one article was not in English (Supplementary Table S3). Two records were retrieved through consultation of the citation. Therefore, 13 records were included in the qualitative analysis. Cohen’s Kappa value for inter-reviewers’ agreement was 0.71 at the title and abstract screening and 0.82 at full-text screening.

### 3.2. Studies and Samples’ Characteristics

Among the 13 RCTs that met the inclusion criteria, two employed a crossover design [23,24], while four adopted a split-mouth design [6,25,26,27]. One study specifically assessed long-term outcomes, evaluating complications occurring eight years after ILA administration [28]. The included studies were published between 2005 and 2023 and were conducted predominantly in the Middle East and Europe, with Saudi Arabia [28,29,30] and Turkey [6,23,25,27] being the most frequently represented countries (Table 1). Funding sources varied considerably across the included studies. Four studies reported institutional or governmental support [6,29,30,31], whereas the majority either declared no external funding or did not provide detailed funding information (Table 1).

The included studies involved a total of 859 enrolled children, since three of them were conducted on the same population [28,29,30], and thus were considered as one. The age of the enrolled children varied between 3 and 13 years, with approximately 51% being female. Sample sizes ranged from 25 to 208 participants (Table 2). Most interventions were performed on primary teeth, with nine studies focusing exclusively on first or second primary mandibular molars [23,24,25,27,28,29,30,31,33] requiring pulpotomy or extraction. One study investigated restorative treatments on permanent teeth [6], while another involved both primary and permanent teeth [34]. In most cases, participants were children offering an adequate collaboration to the proposed treatment evaluated through the Frankl Behaviour Scale (FBS) scoring 3 or 4 (Table 2).

Eleven studies compared ILA, administered using either traditional ILA or CC-ILA delivery systems, with other local anesthesia techniques. These included the IANB [6,24,25,27,28,29,30,31,33] and supraperiosteal anesthesia (SPA) [23,34], administered using either traditional or computer-controlled techniques. Two studies directly compared ILA with CC-ILA [26,32] (Table 2).

The outcomes assessed included pain perception, anesthetic efficacy, and postoperative short- and long-term complications (Table 2) [28,29,30]. Pain perception was assessed using a variety of methods, including behavioral observation of the child’s reactions, such as lack of cooperation attributable to pain [34], and validated pain and behavior scales. These included the Sounds, Eyes, and Motor (SEM) scale [24,27,29,31,32], the Wong–Baker Faces Pain Rating Scale (WBFPRS) [6,23,30], and FBS [33].

Physiological parameters recorded during treatment included heart rate (HR) indicative [6,26,27,31] and peripheral capillary oxygen saturation (SpO_2_) [6], both indicative of the patients’ emotional state. Self-reported pain measures were also collected, including the Visual Analog Scale (VAS) [6,27,33], the Eland Color Scale (ECS) [25], the Visual Numerical Rating Scale (VNRS) [26], and the Maunuksela Faces Pain Scale (FPS) [31,32]. Additionally, in one crossover study and one split-mouth study, children were asked at the end of the observation period to indicate their preference between the two anesthetic procedures [23,25] (Table 2). Anesthetic efficacy was assessed based on procedure duration [33], the need for supplemental injections [26], and failure or delayed onset of anesthesia [34] (Table 2).

Short-term postoperative complications assessed included postoperative pain [23], postoperative hematoma [23], and lip biting within 24 h after treatment [23,31,32,34]. One study evaluated long-term outcomes, specifically the presence of developmental defects of enamel (DDEs) in the permanent dentition 8 years after the administration of local anesthesia in primary teeth [28] (Table 2).

Four studies reported significantly lower pain perception and improved physiological parameters for ILA compared to IANB [25,27,30,31]. Conversely, another four studies found no significant differences between these two local anesthetic techniques [6,24,29,33]. CC-ILA showed superior efficacy compared to SPA [34], but showed inferior performance when compared to CC-SPA [23] (Table 2). When comparing conventional ILA with CC-ILA, the latter appeared to provide greater anesthetic efficacy and lower discomfort [26,32] (Table 2).

Regarding patients’ preference, Öztaş et al. 2005 reported that 68.00% of children preferred CC-ILA compared to 32.00% who preferred IANB [25]. In contrast, Şermet Elbay et al. 2016 found a more even distribution of preferences, with 43.30% favoring CC-ILA and 56.70% favoring CC-SPA [23] (Table 2).

Postoperative complications were reported in four studies. Lip biting was significantly more frequent following IANB or CC-SPA. Helmy et al. 2022 observed a 32.00% incidence with IANB compared with 0% with CC-ILA (*p* < 0.01) [31]. Similarly, Perugia et al. 2017 reported 80.00% occurrence following SPA versus 0% following CC-ILA [34]. In contrast, Şermet Elbay et al. 2016 reported 13.30% with CC-SPA compared with 2.20% with CC-ILA (*p* > 0.05) [23]. Hematoma and postoperative pain were quite similar between CC-ILA and CC-SPA (*p* > 0.05) [23] (Table 2). 

Long-term complications were evaluated in a single study. Baghlaf et al. 2023 reported developmental enamel defects in 11.10% of CC-ILA cases compared to 3.20% with IANB (*p* > 0.05), as shown in Table 2 [28].

### 3.3. Risk of Bias Assessment Results

**Figure 2:**
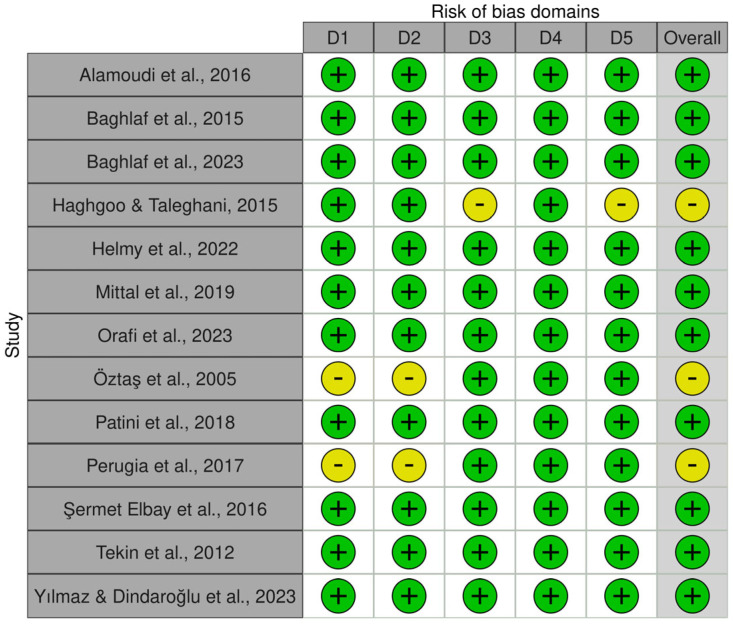
Risk of bias assessment. D1: bias arising from the randomization process, D2: bias due to deviations from intended intervention, D3: bias due to missing data, D4: bias in measurement of the outcome, D5: bias in selection of the reported result. Judgment: green for low risk of bias and yellow for some concerns [6,23,24,25,26,27,28,29,30,31,32,33,34].

**Figure 3:**
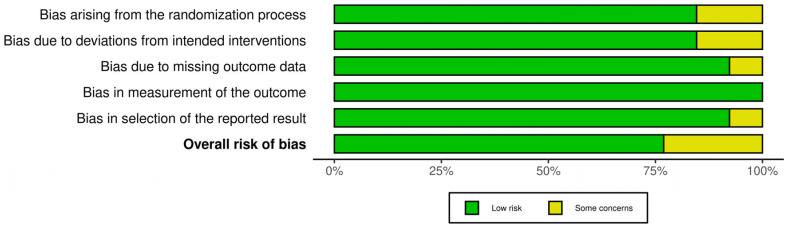
Plot summary of risk of bias assessment.

Risk of bias assessment results are displayed in Figure 2. The assessment indicated an overall low risk across the included studies. Most studies were judged to be at low risk of bias in all five domains. Two studies presented some concerns in domains related to the randomization process and deviations from the intended intervention, which resulted in an overall judgment of “some concerns” [25,34]. Most studies were rated as low risk for bias due to missing outcome data, outcome measurement, and selective reporting, except one study that was judged as having an overall judgment of “some concerns” [24]. No study was assessed as having a high risk of bias in any domain. The summary plot (Figure 3) shows the risk of bias across all domains in the included studies with some concerns in domains regarding the randomization process, deviations from intended interventions, missing outcome data and selection of the reported results.

### 3.4. Meta-Analysis

**Figure 4:**
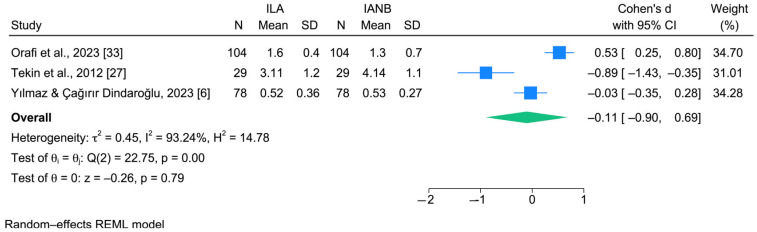
Forest plot showing the pooled effect size (Cohen’s d) for pain perception measured by VAS comparing ILA and IANB [6,27,33].

**Figure 5:**
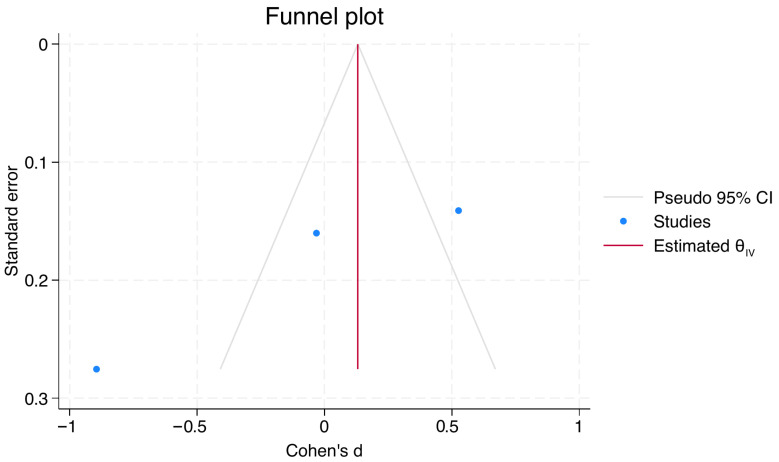
Funnel plot assessing potential publication bias of studies comparing ILA and IANB for pain perception measured by VAS.

The meta-analysis was conducted on only three studies with methodological homogeneity evaluating pain perception using VAS and directly compared ILA with IANB [6,27,33]. A total of 211 participants were included. The forest plot showed that none of the individual studies demonstrated a statistically significant difference in VAS scores between ILA and IANB (Figure 4). The pooled effect estimate did not indicate a significant difference between the two anesthetic techniques (z = −0.26; *p* = 0.79). A high level of heterogeneity was observed among the included studies, with an I^2^ value of 93.24%, suggesting considerable variability in effect estimates. The funnel plot did not reveal a clear asymmetry (Figure 5); however, the small number of included studies limits the reliability of this assessment.

## 4. Discussion

This systematic review aimed to investigate the clinical efficacy, safety, and acceptability of ILA in pediatric patients. The findings indicate that ILA, particularly when delivered via computerized systems, represents an effective, safe, and well-tolerated technique. The included studies consistently reported reduced pain perception, improved stress-related physiological responses, favorable behavioral outcomes, comparable clinical efficacy to traditional anesthetic techniques, and a low incidence of postoperative complications.

In dentistry, as in other areas of medicine, scientific progress is moving toward diagnostic and therapeutic solutions that are increasingly less invasive and more centered on the preferences and needs of patients [35,36]. This shift toward patient-centered and minimally invasive care is particularly important in children, where patient comfort and reduced pain perception can significantly influence treatment acceptance and outcomes. Pain perception using ILA was lower or equal compared to IANB [6,27,33]. Computerized delivery of ILA appears to further reduce nociceptive stimulation [25,26,32]. Several authors attribute these outcomes to the precise control of anesthetic flow rate and volume provided by computerized delivery systems, which allows for a gradual and less traumatic diffusion of the anesthetic solution within the tissues [24,29]. Lower pain scores, combined with reduced escape behaviors, crying, and muscular tension, and improved procedural acceptability highlight the technique’s suitability for pediatric patients. These results contribute to a greater procedural acceptability by the patient with computerized ILA [30].

Physiological parameters such as heart rate and oxygen saturation were generally more stable during ILA and CC-ILA procedures compared to conventional techniques [26,27,31], suggesting lower stress and anxiety levels during anesthesia administration. These findings complement behavioral observations and support the use of CC-ILA as a technique that improves the overall patient experience.

Patient preference data further corroborate previously reported findings, with children often favoring computerized delivery of ILA, with two studies preferring CC-ILA over IANB, and CC-ILA over CC-SPA, respectively [23,25]. Such results underline the importance of patient-centered considerations in pediatric anesthesia and support the integration of CC-ILA in clinical practice to enhance cooperation and satisfaction.

From a behavioral perspective, ILA is associated with more favorable patient behavior during dental procedures, likely due to reduced pain perception. In particular, computerized delivery appears to minimize escape behaviors, crying, and muscular tension, thereby improving clinician–patient interactions and overall treatment outcomes [29,30,31].

The clinical efficacy of traditional or CC-ILA was confirmed across all included studies. Although the technique is primarily limited to single-tooth procedures, none of the included studies reported reduced anesthetic effectiveness. When compared with IANB, ILA and CC-ILA demonstrated equivalent or, in some cases, superior effectiveness [24,26,33], underscoring the importance of selecting the most appropriate anesthetic approach based on the specific procedure and clinical context. Only one included study reported the need for supplemental anesthesia, observing a lower requirement with CC-ILA compared to conventional ILA [26].

Postoperative complications were generally low and mostly limited to minor events using ILA. Lip biting was significantly more frequent following IANB or SPA [31,34], while CC-ILA was associated with minimal soft tissue injury. Hematoma and postoperative pain were comparable between CC-ILA and CC-SPA [23]. Long-term safety was investigated in only one study, which reported no significant developmental defects of enamel in permanent teeth following previous ILA administration in primary molars. While these findings suggest the absence of major long-term adverse effects, the evidence remains limited and preliminary, underscoring the need for well-designed longitudinal studies to confirm long-term safety [28]. In the eight-year follow-up carried out in this study, DDEs were only reported when endodontic treatment had failed, and a related abscess had developed. This data is consistent with dentists’ uncertainty about the actual etiology of DDEs [37].

The risk of bias assessment showed a low risk of bias in most domains, although some domains regarding the randomization process and deviations from intended results raised some concerns. Three studies were conducted on the same population [28,29,30], but evaluated different outcomes and thus bias arising from population level was limited. Sample sizes varied, and the majority of studies focused on primary teeth, particularly mandibular molars, which are commonly treated in pediatric practice. Only a few studies included permanent teeth [23,27,34], suggesting the need for further investigation in this subgroup. Most participants were cooperative children (FBS 3–4), and therefore the findings may primarily reflect outcomes in children able to comply with dental procedures. Accordingly, caution is warranted when extrapolating these findings to less cooperative children, permanent teeth, or more complex clinical situations. Furthermore, the majority of the included studies were conducted in specific geographic regions, particularly the Middle East and Turkey, which may further limit the external validity and global generalizability of the findings of this review.

The decision to limit the meta-analysis to three studies was methodologically justified to ensure clinical homogeneity and meaningful comparability of outcomes. Including studies that assessed different anesthetic techniques or used non-comparable outcome measures would have introduced substantial bias and reduced the interpretability of the pooled estimates. The quantitative synthesis, even if limited to only three studies [6,27,33], did not demonstrate a significant difference in pain perception caused by ILA or IANB.

Although all included studies were RCTs and compared the same anesthetic techniques, a substantial degree of heterogeneity was observed in the absolute VAS values recorded during the injection phase. This heterogeneity may be attributed to subjective factors influencing pain perception, such as the degree of preoperative anxiety and prior dental experience of the children. Moreover, despite using the same pain assessment tool, VAS scores may vary depending on differences in operator technique, injection speed and pressure, needle gauge, and local tissue conditions [4,38]. Nevertheless, subgroup or sensitivity analyses were not feasible due to the limited number of included studies in the meta-analysis. Furthermore, the subjective nature of self-reported pain scales in pediatric populations may contribute to inter-study variability. These considerations likely explain why, although the direction of effect was consistent across studies, the magnitude of VAS scores during injection differed considerably, thereby contributing to the observed heterogeneity in the meta-analysis. These findings suggest that, based on the currently available evidence, both techniques are equivalent in terms of pain reduction as measured by VAS. Further well-designed RCTs using standardized outcome measures are needed to reduce heterogeneity and strengthen the evidence base.

## 5. Conclusions

The present findings indicate that ILA is not associated with greater pain perception during injection when compared with the IANB. When considered alongside existing evidence demonstrating a lower incidence of undesirable soft tissue anesthesia, such as lip biting, and overall efficacy comparable to other techniques, ILA appears to be a particularly suitable anesthetic option for young children.

By providing effective anesthesia without increasing procedural discomfort, ILA may represent a preferable option, particularly in younger or anxious children, as it may contribute to a more positive dental experience and improved patient cooperation. Nevertheless, further research is warranted to investigate its application in additional clinical scenarios, including extractions and restorative procedures on permanent teeth, as well as its use in children with special healthcare needs or dental phobias, to fully elucidate its broader clinical potential.

## Figures and Tables

**Table 1:** Included studies’ characteristics.

Author, Year	Journal	Country	Study Design	Funding
Alamoudi et al., 2016 [29]	Quintessence International	Saudi Arabia	RCT	Deanship of Scientific Research (DSR), King Abdulaziz University, grant n. 5-165/1433
Baghlaf et al., 2015 [30]	Quintessence International	Saudi Arabia	RCT	Deanship of Scientific Research (DSR), King Abdulaziz University, grant n. 1433/15-165
Baghlaf et al., 2023 [28]	Cureus	Saudi Arabia	Follow-up RCT	Not received
Elbay et al., 2016 [23]	Journal of Clinical Pediatric Dentistry	Turkey	Crossover RCT	Not declared
Haghgoo & Taleghani, 2015 [24]	Journal of International Oral Health	Iran	Crossover RCT	Not received
Helmy et al., 2022 [31]	BMC Oral Health	Egypt	RCT	Open access funding from Science, Technology & Innovation Funding Authority (STDF) and Egyptian Knowledge Bank
Mittal et al., 2019 [32]	Anesthesia Progress	India	RCT	Not declared
Orafi et al., 2023 [33]	The Saudi Dental Journal	Libia	RCT	Not declared
Oztas et al., 2005 [25]	Quintessence International	Turkey	Split-mouth RCT	Not declared
Patini et al., 2018 [26]	British Journal of Oral and Maxillofacial Surgery	Italy	Split-mouth RCT	Not declared
Perugia et al., 2017 [34]	European Journal of Paediatric Dentistry	Italy	RCT	Not declared
Tekin et al., 2012 [27]	Journal of International Dental and Medical Research	Turkey	Split-mouth RCT	Not declared
Yılmaz & Çağırır Dindaroğlu, 2023 [6]	Clinical Oral Investigations	Turkey	Split-mouth RCT	Izmir Katip Çelebi University Scientific Research Projects Coordination (Grant n. 2019-TDU-DİŞF-0013)

RCT: randomized controlled trial.

**Table 2:** Main findings of the included studies.

Authors, Year	Procedures	Sample: Number; Age Mean or Range (Years); FBS	Outcome		Results		*p*–Value
Alamoudi et al., 2016 [29]	Pulpotomy of primary lower 2nd molars	N: 91 (30–31–30)		CC-ILA	IANB	CC-IANB	
		FBS: 3–4	SEM during procedure (range)	3.03 ± 0.18; 5.33 ± 2.43	3.35 ± 0.98; 5.52 ± 2.54	3.47 ± 1.01; 5.33 ± 2.38	NS
			Post–op complication	46.70%	35.50%	30.00%	NS
Baghlaf et al., 2015 [30]	Pulpotomy of primary lower 2nd molars	N: 91 (30–31–30)		CC-ILA	IANB	CC-IANB	
		Age: 5–9	Pain–related behavior	0.09 ± 0.11	0.82 ± 0.76	0.45 ± 0.60	<0.01
		FBS: 3–4	PRS	0.13 ± 0.06	1.39 ± 0.20	0.87 ± 0.13	<0.01
Baghlaf et al., 2023 [28]	Pulpotomy of primary lower 2nd molars	N: 40 (9–31)		CC-ILA	IANB		
		Age: 14.9 ± 1.5	DDEs at 8 y follow–up	1 (11.10%)	1 (3.20%)		NS
		FBS: 3–4					
Elbay et al., 2016 [23]	Pulpotomy, restoration, or extraction of primary 1st molars	N: 90 (crossover)		CC-ILA	CC-SPA		
		Age: 6–12	PRS				
		FBS: 3–4	Needle insertion; extraction	+	–		<0.01
			Solution injection; pulpotomy; restoration	+	–		NS
			Post–op pain	26.00%	20.00%		NS
			Post–op hematoma	28.90%	22.20%		NS
			Post–op lip biting	2.20%	13.30%		NS
			Patient preferences	43.30%	56.70%		NS
Haghgoo & Taleghani, 2015 [24]	Pulpotomy of primary lower molars	N: 80		ILA	IANB		
		FBS: 3–4	SEM success rate	88.75	91.25		na
Helmy et al., 2022 [31]	Extraction of primary lower molars	N: 50 (25–25)		CC-ILA	IANB		
		FBS: 3–4	HR				
			Injection	104.64 ± 12.04	113.48 ± 16.66		0.04
			Extraction	107.68 ± 14.33	114.44 ± 19.57		NS
			FPS (positivity)				
			Injection	88.00%	56.00%		<0.01
			Extraction	84.00%	52.00%		<0.01
			SEM				
			Injection	1.15 ± 0.27	2.53 ± 0.88		<0.01
			Extraction	1.76 ± 0.95	2.53 ± 1.10		<0.01
			Post–op lip biting	0.00%	32.00%		<0.01
Mittal et al., 2019 [32]	Extraction of primary molars	N: 82 subjects		CC-ILA	ILA		
		102 procedures	HR				
		(51–51)	Injection	105.70 ± 14.80	101.00 ± 12.00		NS
		Age: 6–13	Extraction	102.00 ± 13.00	109.30 ± 14.50		<0.01
		FBS: 3–4	FPS				
			Injection	1.00	2.00		<0.01
			Extraction	1.00	3.00		<0.01
			SEM				
			Injection	3.00	4.00		<0.01
			Extraction	3.00	5.00		<0.01
Orafi et al., 2023 [33]	Extraction of primary lower molars	N: 208 (104–104)		ILA	IANB		
		Age: 5–13	VAS				
		FBS: na	Injection	1.60 ± 0.40	1.30 ± 0.70		NS
			Extraction	1.80 ± 0.50	1.70 ± 0.60		NS
			FBS	2.20 ± 0.70	2.40 ± 0.30		NS
			Quality of anesthesia	1.40 ± 0.50	1.10 ± 0.40		NS
			Duration extraction	4.80 ± 1.70 min.	4.00 ± 1.20 min.		NS
Oztaş et al., 2005 [25]	Pulpotomy of primary lower 2nd molars	N: 25 (crossover)		CC-ILA	IANB		
		Age: 6–10	ECS				
		FBS: na	Injection	1.40 ± 0.711	2.16 ± 0.75		<0.05
			End of procedure	1.52 ± 1.26	0.48 ± 0.65		<0.05
			Patient preferences	68.00%	32.00%		na
Patini et al., 2018 [26]	Extraction of upper primary molars	N: 76 (crossover)		CC-ILA	ILA		
		Age: 5–12	VNRS (after injection)	4.74 ± 2.80	5.51 ± 2.46		0.04
		FBS: na	HR (after injection)	0.34 ± 7.30	2.72 ± 6.76		0.04
			Need for 2nd injection	6.58%	27.63%		na
Perugia et al., 2017 [34]	Restoration or extractions of primary or permanent molars	N: 50 (25–25)		CC-ILA	SPA		
		FBS: na	Efficacy (from 0′ to 40′)	88.00; 96.00%	56.00; 72.00%		<0.05
			Lack onset anesthesia	0.00–6.67%	13.33–40.00%		na
			Post–op lip biting	0.00%	80.00%		na
			Lost cooperation	0.00%	88.00%		na
Tekin et al., 2012 [27]	Extraction of primary lower 1st molars	N: 29 (crossover)		ILA	IANB		
		Age: 8–9	VAS (injection)	3.11 ± 1.2	4.14 ± 1.1		NS
		FBS: 3–4	SEM				
			Injection	3.83 ± 1.07	5.62 ± 2.13		<0.05
			Extraction	3.93 ± 1.22	5.17 ± 1.89		<0.05
			HR				
			Injection	99.18 ± 18.64	105.18 ± 22.75		NS
			Extraction	104.73 ± 15.10	109.00 ± 14.98		NS
Yılmaz & Çağırır Dindaroğlu, 2023 [6]	Restorative treatments of permanent mandibular 1st molars	N: 78 (crossover)		ILA	IANB		
		Age: 6–12	VAS				
		FBS: 3–4	Injection	0.52 (0.44–0.60)	0.53 (0.47–0.59)		NS
			Procedure	0.57 (0.48–0.65)	0.57 (0.50–0.65)		NS
			PRS during procedure	0.57 (0.49–0.65)	0.60 (0.52–0.67)		NS
			HR				
			Injection	0.60 (0.54–0.67)	0.50 (0.44–0.57)		NS
			Procedure	0.54 (0.47–0.61)	0.49 (0.42–0.56)		NS
			SpO_2_				
			Injection	0.55 (0.48–0.61)	0.50 (0.43–0.57)		NS
			Procedure	0.44 (0.37–0.52)	0.53 (0.46–0.60)		NS

FBS: Frankl Behaviour Scale; N: number; ILA: traditional intraligamentary anesthesia; IANB: traditional inferior alveolar nerve block; CC-ILA: computer-controlled intraligamentary anesthesia; CC-IANB: computer-controlled inferior alveolar nerve block; SPA: supraperiosteal anesthesia; CC-SPA: computer-controlled supraperiosteal anesthesia; SEM: sounds, eyes and motor pain scale; PRS: Wong–Baker Faces Pain Rating Scale; DDEs: developmental defects of enamel; HR: heart rate; FPS: Face Pain Scale from Maunuksela; VAS: Visual Analog Scale; ECS: Eland Color Scale; VNRS: Visual Numerical Rating Scale; SpO_2_: peripheral capillary oxygen saturation; NS: not significant; na: not available.

## Data Availability

All data are included in the manuscript and Supplementary Materials. Additional data are available from the authors upon reasonable request.

## Supplementary Materials

The following supporting information can be downloaded at https://www.mdpi.com/article/10.3390/jcm15051828/s1, Table S1: PRISMA 2020 checklist for systematic reviews and abstract; Table S2: Search strings; Table S3: Excluded records at full-text screening stage.

## Abbreviations

The following abbreviations are used in this manuscript:

LA	Local Anesthesia
IANB	Inferior Alveolar Nerve Block
ILA	Intraligamentary Anesthesia
STA	Single Tooth Anesthesia
VAS	Visual Analog Scale
WBFPRS	Wong–Baker Faces Pain Rating Scale
FPS-R	Faces Pain Scale–Revised
NRS	Numeric Rating Scale
CCLAD	Computer-Controlled Local Anesthetic Delivery
RCTs	Randomized Controlled Trials
CC-ILA	Computer-Controlled Intraligamentary Anesthesia
SPA	Supraperiosteal Anesthesia
FBS	Frankl Behavior Scale
HR	Heart Rate
SpO_2_	Peripheral Capillary Oxygen Saturation
ECS	Eland Color Scale
VNRS	Visual Numerical Rating Scale
DDEs	Developmental Defects of Enamel
CC-SPA	Computer-Controlled Supraperiosteal Anesthesia
