# Supplementary material for: Intraligamentary Anesthesia in Pediatric Patients: Is It an Effective Technique? A Systematic Review and Meta-Analysis
Source: J Clin Med. 2026 Feb 27;15(5):1828. doi: 10.3390/jcm15051828 (PMC12985614; doi:10.3390/jcm15051828)
Supplement: Supplementary file 1 [file jcm-15-01828-s001.zip › jcm-4151130-supplementary.pdf]

Intraligamentary Anesthesia In Pediatric Patients: Is It An Effective Technique? A Systematic Review and Meta-Analysis

Supplementary file

S1. PRISMA 2020 checklist for systematic reviews and abstract

S2. Search strings

S3. Excluded records at full-text screening stage

Table S1. PRISMA 2020 checklist for systematic reviews and abstract.

Section and Topic	Item #	Checklist item	Location where item is reported
**TITLE**			
Title	1	Identify the report as a systematic review.	Page 1
**ABSTRACT**			
Abstract	2	See the PRISMA 2020 for Abstracts checklist.	
**INTRODUCTION**			
Rationale	3	Describe the rationale for the review in the context of existing knowledge.	Page 1-3
Objectives	4	Provide an explicit statement of the objective(s) or question(s) the review addresses.	Page 4
**METHODS**			
Eligibility criteria	5	Specify the inclusion and exclusion criteria for the review and how studies were grouped for the syntheses.	Page 5
Information sources	6	Specify all databases, registers, websites, organisations, reference lists and other sources searched or consulted to identify studies. Specify the date when each source was last searched or consulted.	Page 5
Search strategy	7	Present the full search strategies for all databases, registers and websites, including any filters and limits used.	Supplementary table S2
Selection process	8	Specify the methods used to decide whether a study met the inclusion criteria of the review, including how many reviewers screened each record and each report retrieved, whether they worked independently, and if applicable, details of automation tools used in the process.	Page 5
Data collection process	9	Specify the methods used to collect data from reports, including how many reviewers collected data from each report, whether they worked independently, any processes for obtaining or confirming data from study investigators, and if applicable, details of automation tools used in the process.	Page 5
Data items	10a	List and define all outcomes for which data were sought. Specify whether all results that were compatible with each outcome domain in each study were sought (e.g. for all measures, time points, analyses), and if not, the methods used to decide which results to collect.	Page 4-5
	10b	List and define all other variables for which data were sought (e.g. participant and intervention characteristics, funding sources). Describe any assumptions made about any missing or unclear information.	Page 4
Study risk of bias assessment	11	Specify the methods used to assess risk of bias in the included studies, including details of the tool(s) used, how many reviewers assessed each study and whether they worked independently, and if applicable, details of automation tools used in the process.	Page 6
Effect	12	Specify for each outcome the effect measure(s) (e.g. risk ratio,	Page 6

Section and Topic	Item #	Checklist item	Location where item is reported
measures		mean difference) used in the synthesis or presentation of results.	
Synthesis methods	13a	Describe the processes used to decide which studies were eligible for each synthesis (e.g. tabulating the study intervention characteristics and comparing against the planned groups for each synthesis (item #5)).	Page 6
	13b	Describe any methods required to prepare the data for presentation or synthesis, such as handling of missing summary statistics, or data conversions.	Page 6
	13c	Describe any methods used to tabulate or visually display results of individual studies and syntheses.	Page 5-6
	13d	Describe any methods used to synthesize results and provide a rationale for the choice(s). If meta-analysis was performed, describe the model(s), method(s) to identify the presence and extent of statistical heterogeneity, and software package(s) used.	Page 6
	13e	Describe any methods used to explore possible causes of heterogeneity among study results (e.g. subgroup analysis, meta-regression).	N.A
	13f	Describe any sensitivity analyses conducted to assess robustness of the synthesized results.	N.A
Reporting bias assessment	14	Describe any methods used to assess risk of bias due to missing results in a synthesis (arising from reporting biases).	Page 6
Certainty assessment	15	Describe any methods used to assess certainty (or confidence) in the body of evidence for an outcome.	N.A
**RESULTS**			
Study selection	16a	Describe the results of the search and selection process, from the number of records identified in the search to the number of studies included in the review, ideally using a flow diagram.	Page 7
	16b	Cite studies that might appear to meet the inclusion criteria, but which were excluded, and explain why they were excluded.	Supplementary table S3- Page 7
Study characteristics	17	Cite each included study and present its characteristics.	Page 7-8, Tables 1,2
Risk of bias in studies	18	Present assessments of risk of bias for each included study.	Page 9
Results of individual studies	19	For all outcomes, present, for each study: (a) summary statistics for each group (where appropriate) and (b) an effect estimate and its precision (e.g. confidence/credible interval), ideally using structured tables or plots.	Table 2
Results of syntheses	20a	For each synthesis, briefly summarise the characteristics and risk of bias among contributing studies.	Pages 8-9
	20b	Present results of all statistical syntheses conducted. If meta-	Page 9

Section and Topic	Item #	Checklist item	Location where item is reported
		analysis was done, present for each the summary estimate and its precision (e.g. confidence/credible interval) and measures of statistical heterogeneity. If comparing groups, describe the direction of the effect.	
	20c	Present results of all investigations of possible causes of heterogeneity among study results.	Page 9
	20d	Present results of all sensitivity analyses conducted to assess the robustness of the synthesized results.	N.A
Reporting biases	21	Present assessments of risk of bias due to missing results (arising from reporting biases) for each synthesis assessed.	Page 9
Certainty of evidence	22	Present assessments of certainty (or confidence) in the body of evidence for each outcome assessed.	N.A
**DISCUSSION**			
Discussion	23a	Provide a general interpretation of the results in the context of other evidence.	Page 10
	23b	Discuss any limitations of the evidence included in the review.	Page 11
	23c	Discuss any limitations of the review processes used.	Page 10-11
	23d	Discuss implications of the results for practice, policy, and future research.	Page 10-11
**OTHER INFORMATION**			
Registration and protocol	24a	Provide registration information for the review, including register name and registration number, or state that the review was not registered.	Page 4
	24b	Indicate where the review protocol can be accessed, or state that a protocol was not prepared.	Page 4
	24c	Describe and explain any amendments to information provided at registration or in the protocol.	N.A
Support	25	Describe sources of financial or non-financial support for the review, and the role of the funders or sponsors in the review.	Page 13
Competing interests	26	Declare any competing interests of review authors.	Page 13
Availability of data, code and other materials	27	Report which of the following are publicly available and where they can be found: template data collection forms; data extracted from included studies; data used for all analyses; analytic code; any other materials used in the review.	Page 16
**PRISMA checklist for Abstract**			

Section and Topic	Item #	Checklist item	Reported (Yes/No)
**TITLE**			
Title	1	Identify the report as a systematic review.	Yes
**BACKGROUND**			

Section and Topic	Item #	Checklist item	Location where item is reported
Objectives	2	Provide an explicit statement of the main objective(s) or question(s) the review addresses.	Yes
**METHODS**			
Eligibility criteria	3	Specify the inclusion and exclusion criteria for the review.	Yes
Information sources	4	Specify the information sources (e.g. databases, registers) used to identify studies and the date when each was last searched.	Yes
Risk of bias	5	Specify the methods used to assess risk of bias in the included studies.	Yes
Synthesis of results	6	Specify the methods used to present and synthesise results.	Yes
**RESULTS**			
Included studies	7	Give the total number of included studies and participants and summarise relevant characteristics of studies.	Yes
Synthesis of results	8	Present results for main outcomes, preferably indicating the number of included studies and participants for each. If meta-analysis was done, report the summary estimate and confidence/credible interval. If comparing groups, indicate the direction of the effect (i.e. which group is favoured).	Yes
**DISCUSSION**			
Limitations of evidence	9	Provide a brief summary of the limitations of the evidence included in the review (e.g. study risk of bias, inconsistency and imprecision).	Yes
Interpretation	10	Provide a general interpretation of the results and important implications.	Yes
**OTHER**			
Funding	11	Specify the primary source of funding for the review.	Yes
Registration	12	Provide the register name and registration number.	Yes

Page, M.J.; McKenzie, J.E.; Bossuyt, P.M.; Boutron, I.; Hoffmann, T.C.; Mulrow, C.D.; Shamseer, L.; Tetzlaff, J.M.; Akl, E.A.; Brennan, S.E.; et al. The PRISMA 2020 Statement: An Updated Guideline for Reporting Systematic Reviews. *BMJ* **2021**, *372*, n71. https://doi.org/10.1136/BMJ.N71.

Table S2. Search strings.

**PubMed**	(intraligamentary[tiab] OR intraligamental[tiab] OR "periodontal ligament"[tiab] OR "periodontal injection"[tiab] OR PDL[tiab] OR CCLAD[tiab] OR Wand[tiab] OR "computer controlled"[tiab]) AND ("Pediatric Dentistry"[MeSH] OR child[MeSH] OR adolescent[MeSH] OR pediatr*[tiab] OR child*[tiab] OR adolescen*[tiab]) AND ("Anesthesia, Dental"[MeSH] OR "Anesthesia, Local"[MeSH])
**Embase**	(intraligamentary:ti,ab OR intraligamental:ti,ab OR 'periodontal ligament':ti,ab OR 'periodontal injection':ti,ab OR pdl:ti,ab OR cclad:ti,ab OR wand:ti,ab OR 'computer controlled':ti,ab) AND ('pediatric dentistry'/exp OR 'child'/exp OR 'adolescent'/exp OR pediatr*:ti,ab OR child*:ti,ab OR adolescen*:ti,ab) AND 'dental anesthesia'/exp
**Scopus**	TITLE-ABS-KEY (intraligamentary OR intraligamental OR "periodontal injection" OR "PDL injection" OR "periodontal anesthesia"OR Wand OR "computer controlled anesthesia" OR CCLAD) AND TITLE-ABS-KEY (pediatric OR paediatric OR child OR children OR adolescent) AND TITLE-ABS-KEY (dentistry OR dental)

Table S3. Excluded records at full-text screening stage.

Title	Authors	Year	Journal	Reason for exclusion
A prospective randomized trial of different supplementary local anesthetic techniques after failure of inferior alveolar nerve block in patients with irreversible pulpitis in mandibular teeth	Kanaa, M.D. and Whitworth, J.M. and Meechan, J.G.	2012	Journal of Endodontics	Adult population
A survey of pain, pressure, and discomfort induced by commonly used oral local anesthesia injections.	Kaufman, E. and Epstein, J.B. and Naveh, E. and Gorsky, M. and Gross, A. and Cohen, G.	2005	Anesthesia Progress	Adult population
Comparative evaluation of the efficacy of intraligamentary and supraperiosteal injections in the extraction of maxillary teeth: A randomized controlled clinical trial	Mokshi Jain, Nabeel Nazar	2018	The Journal of Contemporary Dental Practice	Adult population
Subjective pain response to two anesthetic systems in dental surgery: traditional syringe vs. a computer controlled delivery system	Patini, R., Coviello, V., Raffaelli, L., Manicone, P.F., Dehkhargani, S.Z., Verdugo, F., Perfetti, G., D'Addona, A.	2012	Journal of Biological Regulators and Homeostatic Agents	Adult population
Pain during primary molar local anaesthesia with SleeperOne5 computerized device versus conventional syringe: A randomized, split-mouth, crossover, controlled trial	Muller-Bolla, M. and Aïem, E. and Joseph, C. and Davit-Béal, T. and Marquillier, T. and Esclassan, E. and Delfosse, C. and Lopez, S. and Velly, A.M.	2024	International Journal of Paediatric Dentistry	Not used ILA
The assessment of pain sensation during local anesthesia using a computerized local anesthesia (Wand) and a conventional syringe	Ram D, Peretz B.	2003	Journal of Dentistry for Children	ILA with conscious sedation
Assessment of a palatal approach-anterior superior alveolar (P-ASA) nerve block with the Wand® in paediatric dental patients	Ram, D. and Kassirer, J.	2006	International Journal of Paediatric Dentistry	ILA with conscious sedation
Evaluation of Intraligamentous and Intraosseous Computer-Controlled Anesthetic Delivery Systems in Pediatric Dentistry: A Randomized Controlled Trial	Prol Castelo A, García Mato E, Varela Aneiros I, Sande López L, Outumuro Rial M, Abeleira Pazos MT, Rivas Mundiña B, Limeres Posse J.	2022	Children (Basel)	Lack of randomization
Application of computer-controlled local anesthetic delivery system in children	Zhao, X. and Liu, H. and Qin, M.	2011	Hua Xi Kou Qiang Yi Xue Za Zhi	Not in English
Pain behaviour and distress in children during two sequential dental visits: Comparing a computerised anaesthesia delivery system and a traditional syringe	Versloot J, Veerkamp JS, Hoogstraten J.	2008	British Dental Journal	Not stratified data per anesthesia technique
Computerized anesthesia delivery system vs. traditional syringe: Comparing pain and pain-related behavior in children	Versloot J, Veerkamp JS, Hoogstraten J.	2005	European Journal of Oral Sciences	Not stratified data per anesthesia technique
Comparative study of two different computer-controlled local anesthesia injection systems in children: a randomized clinical trial	Abou Chedid JC, Salameh M, El Hindy C, Kaloustian MK, El Hachem C.	2023	European Archives of Paediatric Dentistry	Not used ILA

Comparison of computer controlled local anesthetic delivery and traditional injection regarding disruptive behaviour, pain, anxiety and biochemical parameters: a randomized controlled trial	Anil Ö, Keskin G.	2024	Journal of Clinical Pediatric Dentistry	Not used ILA
The influence of distinct techniques of local dental anesthesia in 9- to 12-year-old children: randomized clinical trial on pain and anxiety	de Camargo Smolarek Pda Silva LMartins Pda Cruz Hartman KBortoluzzi MChibinski A	2021	Clinical Oral Investigations	Not used ILA
Evaluation of efficacy of computer-controlled local anaesthetic delivery system vs traditional injection system for minor pediatric surgical procedures in children	Dempsy Chengappa MM, Prashanth AK.	2022	Medical Journal of Armed Forces of India	Not used ILA
Metallic syringe versus electronically assisted injection system: A comparative clinical study in children	El Hachem, C. and Kaloustian, M.K. and Cerutti, F. and Chedid, N.R.	2019	European Journal of Paediatric Dentistry	Not used ILA
Pain-related behaviour in children: A randomised study during two sequential dental visits	Hembrecht, E.J. and Nieuwenhuizen, J. and Aartman, I.H.A. and Krikken, J. and Veerkamp, J.S.J.	2013	European Archives of Paediatric Dentistry	Not used ILA
Comparing the onset of maxillary infiltration local anaesthesia and pain experience using the conventional technique vs. the Wand in children	Kandiah, P. and Tahmassebi, J.F.	2012	British Dental Journal	Not used ILA
Is it the injection device or the anxiety experienced that causes pain during dental local anaesthesia?	Ozgur Onder Kuscu & Serap Akyuz	2008	International Journal of Paediatric Dentistry	Not used ILA
Evaluation of pain, disruptive behaviour and anxiety in children aging 5-8 years old undergoing different modalities of local anaesthetic injection for dental treatment: a randomised clinical trial	Smolarek P, da Silva L, Martins P, Hartman K, Bortoluzzi M, Chibinski A	2020	Acta Odontologica Scandinavica	Not used ILA
A comparison of pain and anxiety associated with the administration of maxillary local analgesia with Wand and conventional technique.	Tahmassebi, J.F. and Nikolaou, M. and Duggal, M.S.	2009	European Archives of Paediatric Dentistry	Not used ILA
Local anesthesia with SleeperOne S4 computerized device vs traditional syringe and perceived pain in pediatric patients: a randomized clinical trial	Vitale MC, Gallo S, Pascadopoli M, Alcozer R, Ciuffreda C, Scribante A.	2023	Journal of Clinical Pediatric Dentistry	Not used ILA
Behavioral response and pain perception to computer controlled local anesthetic delivery system and cartridge syringe	Yogesh Kumar, T.D. and Baby John, J. and Asokan, S. and Geetha Priya, P.R. and Punithavathy, R. and Praburajan, V.	2015	Journal of Indian Society of Pedodontics and Preventive Dentistry	Not used ILA
